# Clinical Characteristics, Outcomes, and Distribution Patterns of Pathogens Causing Respiratory Infections in Lung Retransplant Recipients

**DOI:** 10.3390/antibiotics14090927

**Published:** 2025-09-13

**Authors:** Min Han, Jae Hoon Kim, Ala Woo, Song Yee Kim, Young Ho Yang, Ha Eun Kim, Jin Gu Lee, Moo Suk Park, Su Jin Jeong

**Affiliations:** 1 Division of Infectious Diseases, Department of Internal Medicine, Yonsei University College of Medicine, Seoul 03722, Republic of Korea; hanmin2025@yuhs.ac (M.H.); jaehoon0215@yuhs.ac (J.H.K.); 2 Division of Pulmonology and Critical Care Medicine, Department of Internal Medicine, Yonsei University College of Medicine, Seoul 03722, Republic of Korea; alwoo@yuhs.ac (A.W.); dobie@yuhs.ac (S.Y.K.); pms70@yuhs.ac (M.S.P.); 3 Department of Thoracic and Cardiovascular Surgery, Yonsei University College of Medicine, Seoul 03722, Republic of Korea; yang321@yuhs.ac (Y.H.Y.); gracehn@yuhs.ac (H.E.K.); csjglee@yuhs.ac (J.G.L.)

**Keywords:** lung retransplantation, respiratory infection, bronchoalveolar lavage, microbiological profile, clinical outcomes

## Abstract

*Introduction*: With advances in surgical techniques and immunosuppressive therapies, lung retransplantation has become a viable option for patients experiencing graft failure. However, retransplantation is associated with inferior clinical outcomes, and infection remains a leading cause of morbidity and mortality in lung retransplant recipients. *Objectives*: This study examined clinical characteristics, outcomes, and microbial spectra of respiratory infections in lung retransplant recipients. *Methods*: This retrospective case–control study, conducted at two tertiary care centers, included 10 lung retransplant patients and 20 matched primary lung transplant patients. Respiratory pathogens identified using bronchoalveolar lavage (BAL) were compared between two groups over a two-year post-transplantation period. *Results*: Pulmonary hypertension was more prevalent in the retransplant group (*p* = 0.030). Five-year mortality and infection-related mortality were higher in the retransplant group (both *p* = 0.015), along with longer hospital and intensive care unit stays (*p* = 0.035 and 0.017, respectively). BAL cultures revealed distinct temporal patterns: *Elizabethkingia* predominated early (31.6% within 1 month) in the retransplant group, with *Pseudomonas* increasing gradually. The primary transplant group demonstrated a more heterogeneous distribution, with *Acinetobacter*, *Pseudomonas*, and *Enterococcus* detected early. *Conclusions*: Retransplant recipients exhibited worse clinical outcomes and a distinct temporal distribution of respiratory pathogens. Particularly, the high incidence of *Elizabethkingia* in lung retransplant recipients highlights the need for center-specific infection surveillance and tailored preventive strategies to improve retransplantation outcomes.

## 1. Introduction

Lung transplantation (LT) is a life-saving therapeutic modality for patients with end-stage pulmonary disease. Despite considerable advancements in surgical techniques, immunosuppressive therapies, and perioperative care, infections remain the leading cause of morbidity and mortality in lung transplant recipients. Respiratory infections are particularly critical owing to the direct involvement of transplanted organs [1,2].

According to the International Society for Heart and Lung Transplantation (ISHLT) Registry, a total of 69,861 lung transplantations were reported worldwide between 1992 and 2025. Since 2000, more than 1000 procedures have been performed annually, increasing to over 2500 per year since 2014, and currently exceeding 3500 cases annually.

With improvements in survival after the initial LT, a subset of patients undergoes a second LT. Within the ISHLT registry, 1597 adult first-time lung retransplant recipients were identified between 2005 and 2017. During this period, between 138 and 188 retransplantations were reported annually, representing approximately 4% to 6% of all lung transplants [1,2,3]. However, lung retransplantation is generally associated with worse clinical outcomes owing to factors such as increased immunological sensitization, accumulated comorbidities, and altered airway environments [4]. Data on infectious complications in this population remain limited, particularly regarding the microbiological landscape and clinical prognosis of respiratory infections. As these patients are often more vulnerable to infection, optimizing infection control strategies is crucial in this population.

The timeline of infection plays a key role in prognosis after transplantation. Previous studies have reported that approximately 75% of infectious episodes occur within the first year after LT, with nearly 42% occurring within the first three months [5,6]. Notably, respiratory infections within the first month of LT have been linked to increased mortality. Thus, analyzing infection patterns based on post-transplantation timelines can provide critical insights into risk periods and guide preventive strategies [1,7].

The objective of this study was to compare the clinical characteristics, survival outcomes, and respiratory pathogens among the primary transplant recipients and retransplant recipients according to the timeline after transplantation. By analyzing the timing and pathogens involved during the first two years, we aimed to identify key differences that may inform future clinical strategies and improve outcomes in this high-risk population.

## 2. Results

### 2.1. Baseline Characteristics

The baseline characteristics are summarized in Table 1. Two groups were similar in age, sex distribution, and body mass index (BMI). The prevalence of pulmonary arterial hypertension was significantly higher in retransplant patients than in primary transplant patients (30.0% vs. 0.0%; *p* = 0.030). Other underlying pulmonary diseases, comorbidities, and immunosuppressive regimens were similar between the two groups.

### 2.2. Clinical Outcomes

**Figure 1:**
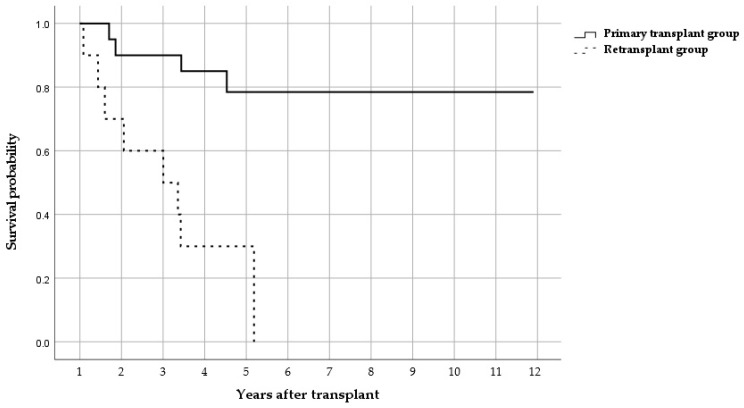
Kaplan–Meier curve for the primary transplant patients and retransplant patients.

Clinical outcomes are summarized in Table 2. The retransplant group had a significantly higher 5-year mortality (70% vs. 20%, *p* = 0.015) and infection-related mortality (70% vs. 20%, *p* = 0.015), while early mortality rates (30-day, 90-day, and 1-year) were not statistically different. The hospital stay (106 vs. 38 days; p = 0.035) and ICU stay (23 vs. 8 days; p = 0.017) were both longer in the retransplant group. Kaplan–Meier curves confirmed the significantly lower survival in retransplant patients (Figure 1).

### 2.3. Microbiological Distribution of BAL Isolates

The microbiological profiles of BAL isolates are summarized in Table 3. Overall, 239 isolates were identified across predefined time intervals.

In retransplant patients, Gram-negative organisms predominated throughout the follow-up. Notably, *Elizabethkingia* was the dominant early isolate, accounting for 31.6% within the first month—a rare finding in primary lung transplantation. During 1–6 months, both *Elizabethkingia* (45.0%) and *Pseudomonas* (28.3%) became increasingly prevalent. By 6–12 months, *Pseudomonas* had overtaken *Elizabethkingia* (36.2% vs. 21.3%, respectively), and by 12–24 months, *Pseudomonas* was clearly the leading pathogen (52.9%). This temporal shift—from the early *Elizabethkingia* predominance to later *Pseudomonas* dominance—represents the distinctive microbiological pattern of retransplant recipients.

In contrast, the primary transplant group showed a more heterogeneous profile. Within the first month, *Acinetobacter* (20.0%), *Pseudomonas* (10.0%), and *Klebsiella* (3.3%) were most common. From 1 to 6 months, Gram-positive cocci, particularly *Enterococcus* (42.1%), predominated. Over time, the total number of isolates decreased without the clear predominance of any single pathogen. Fungal isolates were detected sporadically in both groups and more frequently within the first three months.

## 3. Discussion

In the current study, patients undergoing lung retransplantation had markedly worse clinical outcomes than those receiving their primary transplantation, including higher mortality, longer hospitalization, and a longer ICU stay. The 1- and 5-year survival rates in retransplant recipients were 70% and 30%, respectively, which are lower than the primary transplant recipients.

### 3.1. Survival Outcomes

Previous studies have shown considerable variability in retransplantation outcomes, with 1-year survival rates ranging from 38% to as high as 80% and 5-year survival rates reaching up to 60% in selected cohorts [3,8,9,10,11]. The relatively poor survival in our cohort may be attributable to several unfavorable factors: One patient underwent single-lung retransplantation; three patients underwent retransplantation within two years of their initial lung transplant, a factor previously associated with a worse prognosis; and three patients underwent retransplantation before 2010, an era associated with inferior survival due to less refined surgical techniques and immunosuppressive strategies [3,12].

### 3.2. Microbiological Trends and Clinical Implication

A key novel finding of our study is the unique distribution and timeline of respiratory pathogens after lung retransplantation. While numerous studies in primary lung transplantation have analyzed pathogen distribution according to post-transplant timelines, no such analyses have been conducted in the setting of retransplantation, where prior research has been largely limited to comparisons of survival outcomes [6,13]. To our knowledge, this is the first study to examine the temporal distribution of respiratory pathogens after lung retransplantation.

In particular, *Elizabethkingia* emerged early, accounting for 31.6% of BAL cultures within the first month and remaining a major pathogen for up to 6 months post-transplantation in retransplant patients. This contrasts sharply with primary transplant patients, where no *Elizabethkingia* were detected.

*Elizabethkingia* is an emerging nosocomial pathogen characterized by the intrinsic resistance to multiple antibiotics, particularly against β-lactams and aminoglycosides [14,15]. *Elizabethkingia* infections, mainly affecting neonates and immunocompromised patients, are associated with high mortality rates of 18–41% [16,17,18]. Unlike many Gram-negative bacteria, *Elizabethkingia* displays a unique resistance profile with few horizontal gene transfers, suggesting its intrinsic multidrug resistance [16,17,19]. Given these features and its link to outbreaks in vulnerable populations, routine environmental surveillance and targeted infection-control strategies may be especially important in transplant ICUs, including those caring for lung retransplant recipients. [16,17,18].

In the primary transplant group, *Acinetobacter*, *Klebsiella*, and *Pseudomonas* were identified as the major early pathogens, followed by an increased presence of *Enterococcus* during the intermediate period. Ultimately, *Pseudomonas* and *Klebsiella* became the most common pathogen within the first year, consistent with large lung transplant cohort studies reporting *Pseudomonas* and Enterobacterales as the leading bacterial cause of respiratory infection in primary transplant recipients [6,20,21].

In our study, *Pseudomonas* also became the dominant pathogen in the retransplant group, starting from one month post-transplantation, highlighting its clinical significance in both primary transplants and retransplants. Given the increasing prevalence of multidrug-resistant *Pseudomonas*, targeted pathogen surveillance and antimicrobial susceptibility testing are warranted in lung retransplant recipients to guide the appropriate empiric and directed therapy [7].

We observed a high infection-related mortality rate of 70% in retransplant patients, highlighting the critical impact of respiratory infections on long-term outcomes. Although we did not perform a multivariate analysis to establish infection as an independent predictor of mortality, the temporal correlation between early pathogen isolation and adverse outcomes supports the need for aggressive surveillance, early empirical therapy tailored to center-specific flora, and the use of prophylactic or pre-emptive regimens guided by BAL findings [22].

### 3.3. Limitations and Future Directions

This study has several limitations. First, the small sample size, particularly in the retransplant group, limits the statistical power and generalizability. In addition, the comparator group was selected by manual matching (sex, age, and transplantation date) rather than propensity score matching, which may introduce residual bias. Second, as mentioned above, we did not distinguish between colonization and infection. Also, because additional BAL procedures were performed in patients with suspected infection or rejection, the number of samples varied among patients. Although the variability in BAL sample numbers is a limitation—reflecting a worse clinical status in some patients—it also highlighted the pathogens most relevant to the outcomes. Future studies should analyze infections on an episode-based level to better define their clinical impact. Third, antimicrobial susceptibility data were not analyzed, which limits the interpretation of treatment adequacy and resistance trends.

An additional point of concern is the underappreciated role of fungal pathogens. While fungal isolates were infrequent overall, they were more commonly detected within the first three months, particularly in retransplant recipients. Fungal organisms were only categorized as Candida vs. ‘other,’ and detection was based solely on BAL cultures. Since invasive pulmonary aspergillosis is rarely diagnosed by culture alone, our approach was not sufficient to capture the true burden of serious mold infections. Advanced diagnostics such as galactomannan assays or molecular methods would be required to address this gap [23].

Despite these limitations, this is one of the first studies to systematically characterize microbiological trends over time in lung retransplant recipients, highlighting their vulnerability to early aggressive Gram-negative infections, particularly by *Elizabethkingia*, and the need for customized infection prevention strategies.

## 4. Materials and Methods

### 4.1. Patient Population and Data Collection

We conducted a retrospective case–control study at two tertiary care centers in the Republic of Korea (Sinchon Severance Hospital and Gangnam Severance Hospital), including one that initiated LT. Using the prospectively maintained LT registry of each center, which contained all 277 adult patients who underwent single or bilateral LTs, including retransplantations, performed between January 2005 and December 2022, we identified all 10 adult patients who underwent lung retransplantation during this period. For comparison, 20 patients who underwent primary LT were selected from the same registries and matched to the retransplant patients in a 1:2 ratio based on sex, age (nearest in years), and the closest calendar date of transplantation.

Demographic characteristics, underlying pulmonary diagnoses, perioperative variables, immunosuppressive regimens, and post-transplantation outcomes were extracted from the registries and verified by reviewing electronic medical records. The primary clinical endpoint was all-cause mortality at 30 days, 90 days, 1 year, and 5 years after the index transplantation. Infection-related mortality was determined by conducting a retrospective review of medical records and death certificates and was defined as death attributable to a documented infectious complication. Secondary outcomes included the lengths of intensive care unit (ICU) and hospital stays.

### 4.2. Bronchoalveolar Lavage Surveillance and Microbiological Analysis

Bronchoalveolar Lavage (BAL) is routinely performed at 1, 3, 6, 12, and 24 months post-transplantation and additionally when clinically indicated, such as in cases of suspected pneumonia, acute rejection, bronchiolitis obliterans, unexplained fever, or new pulmonary infiltrates. All BAL specimens were cultured for bacteria and fungi, according to the guidelines of the Clinical and Laboratory Standards Institute (CLSI) [24,25].

Because BAL, unlike expectorated sputum, yields organisms that more specifically reflect the lower airway microbiota in lung transplant recipients, the majority of BAL isolates, except for occasional oral commensals, are considered clinically actionable; therefore, we did not distinguish colonization from infection. Instead, every microorganism isolated from scheduled or clinically indicated BAL samples was extracted with the SCRAP (Severance Clinical Research Analysis Portal) data-retrieval program and included in the analysis.

The relative distribution of isolates at each predefined time point (≤1 month, 1–6 months, 6–12 months, and 12–24 months) was calculated separately for retransplant patients and primary transplant patients to describe temporal shifts in airway microbiota. Mixed cultures were considered as individual isolates. Antimicrobial susceptibility testing was performed using the VITEK-2 system (bioMérieux, Marcy-l’Étoile, France).

### 4.3. Statistical Analysis

All statistical analyses were performed using IBM SPSS Statistics, version 30.0 (IBM Corp., Armonk, NY, USA). Continuous variables are presented as mean ± SD or median with IQR, depending on the distribution, and were compared using the Mann–Whitney U test for non-parametric data. Categorical variables are expressed as counts (n) and percentages (%) and were compared using the chi-square test or Fisher’s exact test, as appropriate. Kaplan–Meier survival curves were generated to compare overall survival between the primary transplant and retransplant recipients. The log-rank test was employed to assess statistical significance between survival curves. Statistical significance was defined as a two-sided *p*-value < 0.05.

## 5. Conclusions

In conclusion, lung retransplantation is associated with higher mortality and a distinct temporal distribution of respiratory pathogens compared with primary transplantation. To the best of our knowledge, this is one of the first studies to characterize the microbiological dynamics of respiratory infections, specifically in the setting of lung retransplantation. Notably, the early predominance of *Elizabethkingia* represents an unusual and clinically important finding. These results underscore the need for targeted infection surveillance and prevention strategies for this high-risk population.

## Figures and Tables

**Table 1:** Comparing characteristics between primary transplant and retransplant patients.

	Primary Transplant	Retransplant	*p*-Value
	Patients (n = 20)	Patients (n = 10)	
Age	40.77 (12.35)	49.00 (15.16)	0.146
Sex, male	10 (50.0%)	4 (40.0%)	0.709
BMI	20.84 (2.88)	19.21 (2.24)	0.235
Underlying pulmonary diseases			
Idiopahtic pulmonary fibrosis	8 (40.0%)	2 (20.0%)	0.419
Chronic obtructive pulmonary disease	3 (15.0%)	4 (40.0%)	0.181
Acute respiratory ditress syndrome	1 (5.0%)	0	0.667
Tubeculosis	6 (31.6%)	3 (30.0%)	1.000
CPFE	1 (5.0%)	0	1.000
Diffuse panbronchiolitis	2 (10.0%)	0	0.540
Bronchiolitis obliterans	3 (15.0%)	4 (40.0%)	0.181
CTD-ILD	3 (15.0%)	0	0.532
Other ILD	1 (5.0%)	0	1.000
Pulmonary arterial hypertension	0	3 (30.0%)	0.030 *
Others	2 (10.0%)	2 (20.0%)	0.615
Underlying diseases			
Diabetes mellitus	10 (50.0%)	3 (30.0%)	0.440
Hypertension	7 (35.0%)	2 (20.0%)	0.675
Coronary artery occlusive disease	1 (5.0%)	0	1.000
Congestive heart failure	1 (5.0%)	1 (10.0%)	1.000
Cerebral vascular disease	1 (5.0%)	0	1.000
Chronic kidney disease	2 (10.0%)	2 (20.0%)	0.584
Chronic liver disease	1 (5.0%)	0	1.000
Connective tissue disease	3 (15.0%)	0	0.532
Cancer	4 (20.0%)	1 (10.0%)	0.584
Previous stem cell transplantation	3 (15.0%)	2 (20.0%)	1.000
Immunosuppressants			
Tacrolimus	20 (100%)	10 (100%)	-
Mycophenolate	20 (100%)	8 (80.0%)	0.103
Prednisolone	20 (100%)	10 (100%)	-
Cyclosporine	20 (100%)	10 (100%)	-
Azathioprine	0	2 (20.0%)	0.103
Basiliximab	1 (5.0%)	1 (10.0%)	1.000

Abbreviations: BMI, body mass index; CPFE, combined pulmonary fibrosis and emphysema; and ILD, interstitial lung disease. Data are presented as mean ± standard deviation (SD) for continuous variables and n (%) for categorical variables. “-” indicates not applicable because both groups had identical values (100%). Statistically significant values, *p* < 0.05, are indicated with an asterisk *.

**Table 2:** Comparing outcomes between primary transplant and retransplant patients.

	Primary Transplant	Retransplant	*p*-Value
	Patients (n = 20)	Patients (n = 10)	
Overall mortality n (%)			
30 days	0 (0.0%)	1 (10.0%)	0.333
90 days	0 (0.0%)	1 (10.0%)	0.333
1 year	2 (10.0%)	3 (30.0%)	0.300
5 years	4 (20.0%)	7 (70.0%)	0.015 *
Infection-related mortality	4 (20.0%)	7 (70.0%)	0.015 *
Other outcomes (days)			
Length of stay	38 (26–89)	106 (55–188)	0.035 *
ICU stay	8 (5–15)	23 (14–31)	0.017 *

Data are presented as median (interquartile range, IQR) and n (%) for categorical variables. Statistically significant values, *p* < 0.05, are indicated with an asterisk *.

**Table 3:** Distribution of causative microorganisms in respiratory infection of LT recipients.

**BAL at <1 Month Post-Transplantation (n = 49)**	**BAL Isolates in Primary Transplant Patients (n = 30)**		**BAL Isolates in Retransplant Patients (n = 19)**	
	n	%	n	%
Gram-negative species	13	43.3%	11	57.9%
*Escherichia coli*	2	6.7%	3	15.8%
*Klebsiella*	1	3.3%	2	10.5%
Other Enterobacterales	0	-	0	-
*Pseudomonas*	3	10.0%	0	-
*Acinetobacter*	6	20.0%	0	-
*Stenotrophomonas maltophilia*	0	-	0	-
*Elizabethkingia*	0	-	6	31.6%
Other Gram-negative species	1	3.3%	0	-
Gram-positive species	15	50.0%	5	26.3%
Coagulase negative staphylococcus	5	16.7%	2	10.5%
*Staphylococcus aureus*	2	6.7%	0	-
*Streptococcus*	2	6.7%	0	-
*Enterococcus*	3	10.0%	0	-
Other Gram-positive species	3	10.0%	3	15.8%
Fungus	2	6.7%	3	15.8%
*Candida*	1	3.3%	1	5.3%
Others	1	3.3%	2	10.5%
**BAL at 1–6 months post-transplantation (n = 79)**	**BAL isolates in primary transplant patients (n = 19)**		**BAL isolates in retransplant patients (n = 60)**	
	n	%	n	%
Gram-negative species	5	26.3%	48	80.0%
*Escherichia coli*	0	-	0	-
*Klebsiella*	4	21.1%	1	1.7%
Other Enterobacterales	0	-	0	-
*Pseudomonas*	0	-	17	28.3%
*Acinetobacter*	1	5.3%	2	3.3%
*Stenotrophomonas maltophilia*	0	-	0	-
*Elizabethkingia*	0	-	27	45.0%
Other Gram-negative species	0	-	0	-
Gram-positive species	12	63.2%	12	20.0%
Coagulase negative staphylococcus	1	5.3%	1	1.7%
*Staphylococcus aureus*	0	-	0	-
*Streptococcus*	0	-	0	-
*Enterococcus*	8	42.1%	2	3.3%
Other Gram-positive species	3	15.8%	9	15.0%
Fungus	2	10.5%	0	-
*Candida*	2	10.5%	0	-
Others	0	-	0	-
**BAL at 6–12 months post-transplantation (n = 54)**	**BAL isolates in primary transplant patients (n = 7)**		**BAL isolates in retransplant patients (n = 47)**	
	n	%	n	%
Gram-negative species	4	57.1%	32	68.1%
*Escherichia coli*	0	-	0	-
*Klebsiella*	2	28.6%	0	-
Other Enterobacterales	0	-	0	-
*Pseudomonas*	1	14.3%	17	36.2%
*Acinetobacter*	0	-	2	4.3%
*Stenotrophomonas maltophilia*	0	-	2	4.3%
*Elizabethkingia*	0	-	10	21.3%
Other Gram-negative species	1	14.3%	1	2.1%
Gram-positive species	4	57.1%	15	31.9%
Coagulase negative staphylococcus	1	14.3%	8	17.0%
*Staphylococcus aureus*	0	-	0	-
*Streptococcus*	0	-	0	-
*Enterococcus*	0	-	4	8.5%
Other Gram-positive species	3	42.9%	4	8.5%
Fungus	0	-	0	-
*Candida*	0	-	0	-
Others	0	-	0	-
**BAL at 1–2 years post-transplantation (n = 57)**	**BAL isolates in primary transplant patients (n = 6)**		**BAL isolates in retransplant patients (n = 51)**	
	n	%	n	%
Gram-negative species	5	83.3	40	78.4%
*Escherichia coli*	0	-	0	-
*Klebsiella*	1	20.0%	2	3.9%
*Other Enterobacterales*	0	-	6	11.8%
*Pseudomonas*	0	-	27	52.9%
*Acinetobacter*	0	-	0	-
*Stenotrophomonas maltophilia*	0	-	0	-
*Elizabethkingia*	0	-	2	3.9%
*Other Gram-negative species*	3	60.0%	3	5.9%
Gram-positive species	1	20.0%	11	21.6%
*Coagulase negative staphylococcus*	1	20.0%	2	3.9%
*Staphylococcus aureus*	0	-	0	-
*Streptococcus*	0	-	0	-
*Enterococcus*	0	-	0	-
*Other Gram-positive species*	0	-	9	17.6%
Fungus	0	-	0	-
*Candida*	0	-	0	-
Others	0	-	0	-

All other organisms are not detected. “-” indicates no isolates detected. Bacteria were reported at the genus level when species-level identification was not available.

## Data Availability

The data presented in this study are available from the corresponding author on reasonable request.

## Abbreviations

The following abbreviations are used in this manuscript:

LT	Lung transplantation
BAL	Bronchoalveolar lavage
ICU	Intensive care unit
BMI	Body mass index
CTD	Connective tissue disease
ILD	Interstitial lung disease
SD	Standard deviation
IQR	Interquartile range
